# Primary osteosarcoma of the uterus: a report of two cases

**DOI:** 10.4314/gmj.v55i3.10

**Published:** 2021-09

**Authors:** Kofi Effah, Edem Hiadzi, Anita Osabutey, Alex K Boateng, Agyeman B Akosa, Jehoram T Anim

**Affiliations:** 1 Department of Obstetrics &Gynaecology, Catholic Hospital, Battor, Ghana; 2 Lister Hospital & Fertility Centre, Accra, Ghana; 3 Cellular Pathology Laboratory, Ghana Standards Authority, Accra, Ghana

**Keywords:** Osteosarcoma, Uterus, Primary, extraskeletal, Ghana

## Abstract

Primary extraskeletal osteosarcoma is an uncommon disease and has been reported to affect the uterus only rarely. Less than 20 cases have so far been reported in the English literature. The common clinical presentation is heavy bleeding *per vaginam*, and in virtually all cases, the diagnosis has been made at an advanced stage of the disease. Various authors have recommended adjuvant chemotherapy, but outcomes have so far been uniformly poor, with survival extended by months rather than years. We present two cases of this rare condition, which were diagnosed four months apart within our histopathology laboratory andconfirmed the very late presentation of the disease in one and the poor survival of both patients.

**Funding:**

None declared

## Introduction

Uterine sarcomas make up less than 1% of all gynaecologicalneoplasms.^1^ They can be classified as either homologous (consisting of components native to the uterus) or heterologous (consisting of components foreign to the uterus). The majority of uterine sarcomas are homologous, with the heterologous being extremely rare. Extraskeletal osteosarcomas are unusual tumours with a poor prognosis. They account for about 1–2% of all soft tissue tumours and approximately 2–4% of all osteosarcomas^2^ Primary uterine osteosarcoma is an extremely rare gynaecological malignancy. Nineteen cases have so far been reported in the English literature.^3^ We present two cases of this rare entity that were diagnosed in one diagnostic centre in Ghana within four months.

## Case Reports

### Case 1

A 60-year-old Ghanaian woman (para 8), 20 years postmenopausal presented to a district hospital in Ghana with a three-month history of progressively worsening, unprovoked post-menopausal bleeding with associated weight loss. Chest X-ray and other laboratory examinations were normal. Speculum examination revealed blood at the posterior fornix and a necrotic cord-like tissue extending from the endocervical. The uterus was bulky and parametria were free. A pelvic ultrasound showed a bulky anteverted uterus with thickened endometrium.

The endometrial cavity showed a fluid collection with echogenic material seen with the fluid. Pouch of Douglas was free and right and left ovaries were not visualised. Endometrial curettage for histopathological evaluation showed mostly necrotic tumour with small viable areas showing spindle and bizarre-shaped giant malignant cells. An initial pathological diagnosis of malignant mixed Mullerian tumour was suggested, and hysterectomy advised.The patient underwent total abdominal hysterectomy, bilateral salphingo-oopherectomy, and pelvic and para-aortic lymphadenectomy.

***Intra-operative findings:*** The uterus was 16 weeks, globular, soft with serosa broken antero-fundally by the tumour. Ovaries appeared atrophic with normal-looking fallopian tubes. Few enlarged pelvic and para-aortic lymph nodes were seen. The liver, spleen, gall bladder, appendix and small and large bowels all looked and felt normal on palpation. No gross tumour was seen on the omentum or within the peritoneal cavity.

**Figure 1:**
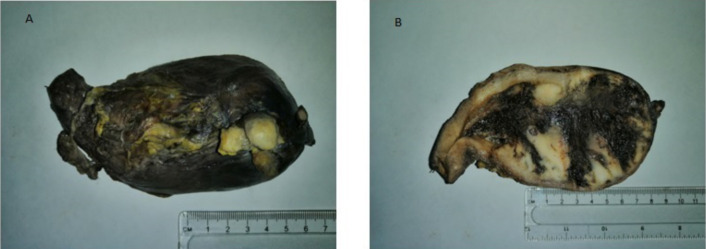
Enlarged uterus (Case 1). A: Outer surface of the uterus shows a small breach of the myometrium at the fundus, revealing a tumour. B: The cut surface of the uterus showing a tumour filling the uterine cavity.

***Pathology:*** A deformed uterus, cervix and both adnexae together weighing 500g. The body of the uterus measured 14x11x10cm, with the cervix measuring 2x2cm. Both left and right ovaries and fallopian tubes were grossly normal. The cut surface of the uterus showed a partly necrotic tan-brown endometrial mass filling the cavity and measuring 10.5cm in maximum dimension (Figure 1).

The mass involved the inner two-thirds of the myometrium and had broken through the myometrium at one point. Left pelvic dissection specimen contained five lymph nodes. Right pelvic dissection specimen contained nine lymph nodes. Para-aortic specimen contained five lymph nodes.

**Figure 2:**
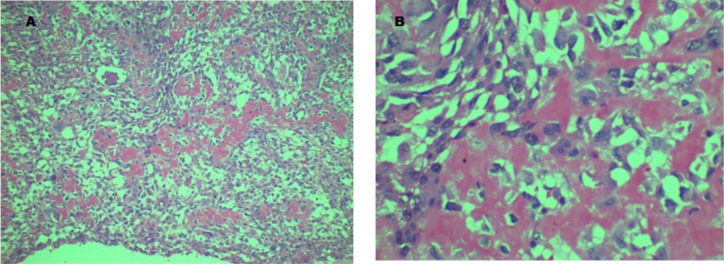
Micrographs of tumour in Case # 1. A: Shows malignant mesenchymal tumour with stromal deposits of osteoid (H&E x100). B: Higher magnification to demonstrate stromal osteoid (H&E x 400).

***Histological examination:*** The uterine mass showed a polypoid and infiltrative, vascular and haemorrhagic tumour, focally transmural at the point of perforation. Tumour was composed of pleomorphic spindle and polygonal and giant malignant cells with areas of malignant, focally calcified osteoid and chondroid deposits (Figure 2).

**Figure 3:**
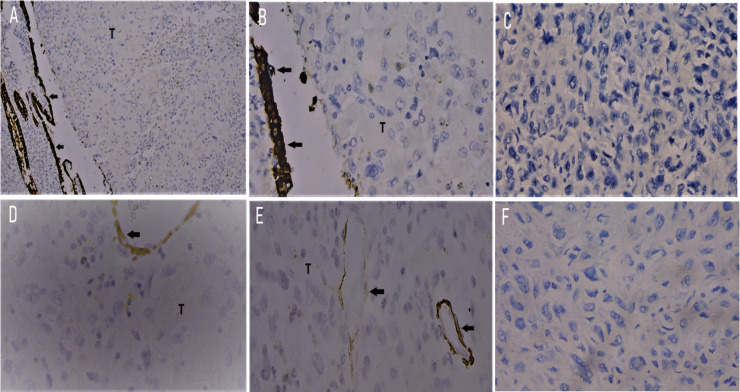
Immunostains. A, B and C: Cytokeratin (AE1/AE3). A: Strong positive staining of normal endometrial epithelium is indicated by arrows. Tumour cells (T) show no staining. (A: x100). B & C: Higher magnification to show no staining of tumour cells (x400). D, E and F: Smooth muscle actin (SMA). D and E show positive smooth muscle cells in the wall of normal blood vessels but no staining of tumour cells (T) (D, E &F; x400).

The features were those of osteosarcoma. No epithelial or other homologous uterine components were seen in the tumour, as supported by negative immunostains for cytokeratin (AE1/AE3) and smooth muscle actin (SMA) (Figure 3). Both ovaries and both fallopian tubes were atrophic. Left and right pelvic and para-aortic lymph nodes showed no metastatic deposits.

***Diagnosis:*** Primary osteosarcoma of the uterus (Stage IC).

After surgery, she was referred for adjuvant chemotherapy at the Centre for Radiation and Nuclear Medicine, Korle Bu Teaching Hospital, but she defaulted due to financial constraints. Review nine months after surgery revealed bilateral opacities in the lung fields. She subsequently developed bilateral pleural effusion and palpable abdominal masses. Twelve months after surgery, an abdominal ultrasound scan showed a complex solid and cystic mass in the mid-abdomen (left periumbilical) measuring 8.17cm x 7.34cm. She died on April 14, 2020, fourteen months after the surgery. No autopsy was done.

### Case 2

A 42-year-old woman first presented in 2009 with lower abdominal pain and was diagnosed as multiple uterine fibroids. Teen year later, she presented with prolonged menstrual flow. Abdominal ultrasound showed an enlarged uterus containing fluid. An abdominal CT scan also showed a left adnexal mass with mild to moderate ascites, hepatomegaly, and left lower anterior abdominal wall mass. She was given Goserelin (Zoladex) for three months to shrink the uterus. CT scan of chest showed nodules in the lung. Laparotomy was done in May 2019.

***Intra-operative findings***: Uterus was 18 week-size, with bilateral cystic ovaries. Ascites was 1.2 litres, and there was a tumour attached to the sigmoid colon, extending to the upper rectum. She underwent total abdominal hysterectomy with bilateral salpingo-oophorectomy, resection of the sigmoid colon and upper rectum with the construction of colostomy, appendicectomy and adhesiolysis.

**Figure 4:**
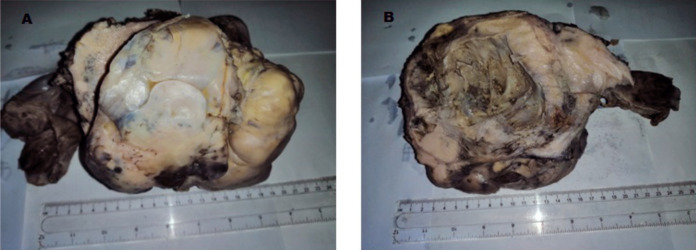
Enlarged, grossly deformed uterus with tumour nodules in Case # 2. **A:** Outer surface of the uterus. **B:** Cut surface showing transmural tumour nodules with cervix replaced by tumour.

***Pathology*** Grossly deformed, multinodular uterus, with the cervix and both adnexa, together weighed 2.1kg. Both right and left ovaries contained tiny cysts but were otherwise unremarkable. Cut surface of uterus showed multiple greyish-white, haemorrhagic subserous, intramural and submucosal nodules, the largest 11cm in diameter (Figure 4). Appendix 6x7cm and a segment of the large bowel with irregular serosal surface were also present in the container.

**Figure 5:**
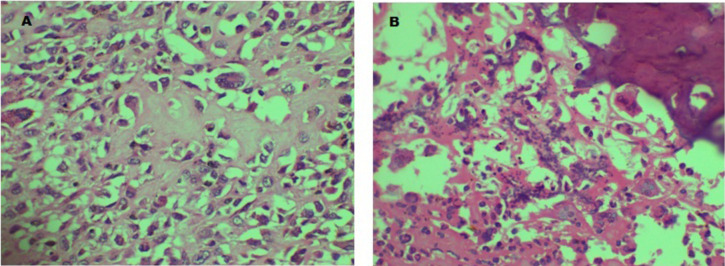
Micrograph of tumour in Case # 2. **A:** Malignant mesenchymal tumour with abundant stromal osteoid deposits. **B:** Tumour showing calcification of osteoid (A & B; H&E x 400).

***Histological examination:*** The uterine nodules were tumour composed of pleomorphic spindle and polygonal malignant cells with scattered tumour giant cells. Mitoses were frequent, and there were areas of osteoid deposits with tumour bone formation (Figure 5).

The tumour had traversed the myometrium in areas to form serosal deposits. Cervix had been replaced by tumour tissue, and tumour deposits were also present in the appendix and large bowel serosa. Ovaries and fallopian tubes were unremarkable. No epithelial or other homologous uterine components were seen in the tumour as confirmed by negative immunostains for cytokeratin (AE1/AE3) and smooth muscle actin (SMA) (Figure 3).

***Diagnosis:*** Primary osteosarcoma of the uterus (Stage IVB)

Post-operatively, she developed pulmonary embolism, chest infection, hypertension, and anaemia, which were managed conservatively. She was discharged on postoperative Day 16. She was re-admitted a month later with severe anaemia and urinary tract infection for which she received appropriate treatment. CT scan of chest showed multiple pleural and pulmonary metastases, nodular deposits in anterior epicardial fat, mediastinal and extrathoracic lymphadenopathy, but no pleural or pericardial effusion and no thoracic bony lesions. Pelvic and abdominal CT-scan showed complex solid/cystic lobulated mass arising from the pelvis and multiple metastases involving retroperitoneum, anterior abdominal wall, and along the colostomy site subcutaneously. No osseous metastatic deposits or pathological fracture was noted. The bladder was encased in the tumour, with moderate left obstructive uropathy.

The patient received five cycles of radiotherapy during which she developed intestinal obstruction, with large left paracolic gutter and right lumbar intra-abdominal masses causing mass effect on adjacent structures. Chemotherapy was recommended, but she went rapidly downhill and died four months after the initial surgical intervention. No autopsy was done.

## Discussion

Primary osteosarcoma of the uterus is a rare tumour. The earliest report of a case in English literature was by Stier and Lyman in 1936.^4^ Because of its rarity, only individual case reports are available in the literature.^5^–^13^ Successive authors have reviewed available case reports to better define the tumour in terms of diagnosis, management and prognosis. We are fortunate to present two cases of the tumour diagnosed in the same pathology laboratory within four months of each other in two subjects with different stages of the disease.

The most reliable diagnostic tool is the microscopic examination of H&E-stained sections of the tumour. Histological parameters include features of a malignant mesenchymal neoplasm, with osteoid formation, lacking an epithelial component,^12^ confirmed with appropriate epithelial and mesenchymal immunohistochemical stains.^12^ In our two cases, the mesenchymal nature and osteoid components were evident on routine H&E staining. Absence of epithelial components was confirmed by examining multiple sections and negative immunostaining for cytokeratin. Staining for smooth muscle actin (SMA) was alsonegative in the tumour cells, indicating that these two cases were not examples of the rare osteoid metaplasia in pleomorphic leiomyosarcoma. Some workers have suggested that the histological origin of uterine osteosarcomas may be secondary to either mesenchymal differentiation or a smooth muscle neoplasm with malignant osseous metaplasia.^14^

These two cases satisfy the three criteria specified for diagnosis of primary osteosarcoma of the uterus, namely: exclusion of primary bone lesion, presence of neoplastic osteoid and absence of epithelial or other specific homologous uterine elements.^9^

Metastasis from a skeletal osteosarcoma was also excluded in our two patients by absence of skeletal lesions on imaging studies.

Successive reports have indicated that chemotherapy is the most useful adjuvant therapy. Trial of radiotherapy in the second patient with advanced disease proved disappointing, as she demonstrated rapid spread of the tumour within a short time. Unfortunately, the first patient with a relatively early stage of the disease could not afford the chemotherapy treatment recommended. Although suggested as the most appropriate adjuvant therapy, the ideal chemotherapy regimen for primary osteosarcoma of the uterus remains unsettled. Most recommended chemotherapeutic agents are derived from protocols used for the much commoner skeletal osteosarcomas or other soft tissue sarcomas. Despite chemotherapy, the disease has been reported to pursue a rapid course, leading to death within months. The longest surviving patient in the literature lived to 37 months.^9^

Patients diagnosed with primary osteosarcoma of the uterus in the literature were aged over 40 years, as in our two aged 60 and 42 years, respectively. Our 60-year-old patient also gives us an insight into the possible natural progression of the disease without the benefit of adjuvant chemotherapy, from a relatively early stage into a rapidly progressive disease leading to death ten months after diagnosis. Our second patient presented with a more advanced stage of the disease, went rapidly downhill despite radiotherapy and died within four months. These two cases confirme that the disease pursues an aggressive course despite adjuvant therapy.

## Conclusion

Two cases of primary osteosarcoma have been presented, essentially to raise awareness of the condition in older women, as a cause of prolonged bleeding *per vaginam*. The diagnosis was based on histological examination of the tumour and the establishment of its primary nature in the uterus, through absence of homologous uterine components in the tumour. Our two cases further emphasise the rapid progression of the disease, resulting in death within months. This paper also highlights in the 60-year-old subject the difficulty cancer patients face in accessing quality health care in poorer countries such as Ghana.
